# Creation and Operation of a COVID-19 Pooled Testing Collection Site Prior to a CTC Rotation

**DOI:** 10.1093/milmed/usab513

**Published:** 2021-12-13

**Authors:** Savannah Smith, Jessica Heffner, Rachael King, Haley Guzman

**Affiliations:** 1st Armored Brigade Combat Team, 1st Infantry Division, Fort Riley, KS 66442, USA; Irwin Army Community Hospital, Fort Riley, KS 66442, USA; Irwin Army Community Hospital, Fort Riley, KS 66442, USA; 1st Armored Brigade Combat Team, 1st Infantry Division, Fort Riley, KS 66442, USA

## Abstract

**Introduction:**

This brief report describes the process, used by the 1st Infantry Division (1ID) and Irwin Army Community Hospital (IACH) at Fort Riley, Kansas, for conducting pooled testing collection of over 2,500 Soldiers prior to a large-scale exercise involving multiple units.

**Materials and Methods:**

The authors captured after action review comments on the process and results of their pooled specimen collection site. Pooled specimen test results were reviewed and classified according to Aberdeen Proving Ground criteria to determine the percentage of successful and failed pooled specimens.

**Results:**

1ID and IACH performed pooled testing collection and shipment of 2,684 specimens divided into 298 pools over 6 flight manifests. Of the 298 pooled specimens, 4 (1.34%) were found to be inconclusive or invalid, and the other 294 (98.7%) had sufficient number of human cells to be certified as SARS-CoV-2 (COVID-19) positive or COVID-19 not detected.

**Conclusion:**

Pooled testing collection is a complex process that may continue to be a requirement for mass screening of COVID-19 prior to military operations. While planning should be tailored to the specific mission and unit, key factors that the authors feel are required for pooled testing to be successful in any situation are standardized training and personnel continuity, quality assurance, administrative oversight by the unit, and collaboration and communication between all involved entities.

## INTRODUCTION

The SARS-CoV-2 (COVID-19) pandemic adds a layer of complexity to normal military operations. Commanders must now balance priorities of protecting the force, preventing the spread, and preserving readiness to fight and win our nation’s wars. In order to do this, Headquarters, Department of the Army (HQDA) required U.S. Army Medical Command to conduct pooled COVID-19 surveillance testing for 1st Infantry Division (1ID) prior to their planned Combat Training Center (CTC) rotation.^1^

Pooled testing was first introduced in the 1940s and is used to detect HIV and hepatitis B and C viruses when screening blood products.^2,3^ The Food and Drug Administration (FDA) approved assays for the detection of SARS-CoV-2 under emergency use authorization (EUA) in the spring of 2021. Several studies have examined whether using a EUA SARS-CoV-2 assay in a public health setting for pooled testing enables organizations impacted by scarcity of key resources to test large numbers of individuals for COVID-19.^3^ One study in India discussed their current pooled testing procedures, which included pipetting out a designated sample size from each viral transport media (VTM) to add to a pool tube, while maintaining individual sample integrity for retesting as an individual sample if the pool test was positive.^4^ Optimal pool size is dependent on multiple factors, including prevalence, test sensitivity, and test specificity, and generally ranges from 5 to 32 individual samples.^4–6^ Specificity can be impacted by the inherent performance of the test, swabbing the wrong patient or placing the wrong sticker on the specimen, virus contamination at any point, and transcription errors when reporting the result.^7^

Irwin Army Community Hospital (IACH) received instructions to conduct pooled testing of over 2,500 Soldiers prior to a CTC rotation in September 2020 using a pool size of 10 and to be prepared to collect and individually run a second swab of all Soldiers with a positive pooled test result. A literature review did not find published guidance on established processes to minimize human error during pooled SARS-CoV-2 specimen collection and transport. This article aims to share the successful process developed by IACH and 1ID to help guide future planning at other military treatment facilities (MTFs).

## MATERIALS AND METHODS

1ID and IACH coordinated a joint effort to complete COVID-19 screening for approximately 3,500 Soldiers prior to their CTC rotation in September 2020. This was initially done through individual testing in each subordinate unit’s area of operations prior to HQDA’s mandate to switch to pool testing. With 5 days’ notice, IACH and 1ID quickly transitioned from individual testing to pool testing using the existing centralized infrastructure of a drive-through specimen collection site at the IACH parking garage. Key processes are highlighted below.

### Personnel, Training, and Organization

IACH and 1ID prioritized safety, quality assurance (QA), and redundancy to determine staffing needs and agreed that 40 individuals were required for successful operations. IACH and 1ID tasked subordinate units to provide a mix of medical and nonmedical personnel to fill the 40 roles and required each individual to work within their assigned role throughout the entire operation (Table I). The IACH Education Department standardized training and supervision of all medics at the specimen collection stations to minimize the risk of human error during specimen collection. One individual, usually the IACH Education Department Head, supervised overall operations, provided on-the-spot corrections and process adjustments, and collected after action review (AAR) comments.

**TABLE I.** Personnel, Training, Equipment, and Organization Used at Drive-through Collection Site. All Personnel Utilized Appropriate Levels of Personal Protective Equipment (PPE) Based on the Duties They Performed and Their Proximity to the Nasopharyngeal Swab Collection

Roles	Personnel	Special training and equipment	Tasks
Traffic Controller	8 nonmedical	Reflective vests Radios	Direct traffic Activate overflow parking site as needed
CAC Collector	3 nonmedical	Reflective vest	Shuttle CAC between POV and admin table Place specimen label under driver-side windshield wiper
Admin	3 MSC officers	Laptops MC4 2D scanners Preprinted labels	Scan CAC and assign label to each patient Give label to CAC Collector Email excel sheet to print station
Specimen Collection	16 medics	Specimen swabs VTM Ice	See Figure 2
QA	2 medics 2 nonmedical 1 medical NCO	Specimen bags Ice	Inspect and sort all specimens into pooled specimen bags Move pooled specimen bags to lab Resupply CLVIII and ice Deconflict issues and adjust operations as needed
Print Station	1 MSC officer	IACH computer and printer access	Receive and print all pooled testing label trackers Pair each tracker with its pooled specimen bag
Lab	1 lab employee 2 medics	Category B Infectious Disease Shipping certification	Receive, process, package, and ship pooled specimen bags to APG
Supervisor	1 MSC officer or civilian leader		Supervise overall operations, provide on-the-spot corrections and adjustments, and collect AAR comments

Abbreviations: Nonmedical—Soldier of any rank and training background. Medic—combat medics of any skill level. MSC officer—Medical Service Corps officer, such as a medical planner. CAC—common access card. QA—quality assurance. NCO—non-commissioned officer. MC4—Medical Communications for Combat Casualty Care. VTM—viral transport media. POV—privately owned vehicle. CLVIII—medical materiel (supplies). AAR—after action review.

### Site Setup and Specimen Flow

**FIGURE 1.**
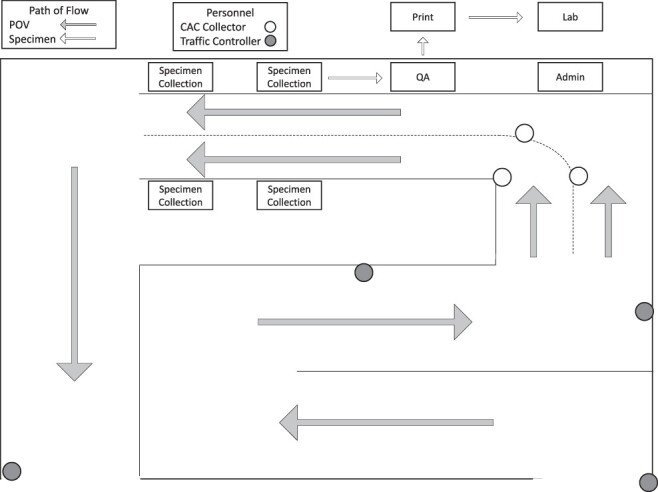
Implementation of drive-through specimen collection using pre-existing hospital parking garage structure and traffic flow, where arrows indicate flow of POVs and specimens throughout the process. POV—privately owned vehicle. CAC—common access card. QA—quality assurance.

The drive-through testing occurred on the ground floor of the IACH parking garage, utilizing existing vehicle flow design (Fig. 1).

Aberdeen Proving Ground (APG) required each patient’s specimen vial to be labeled with a unique contributor sample that was aligned with the patient’s Department of Defense Identification (DoD ID) Number on an accompanying excel sheet.^8^ IACH and 1ID preprinted specimen labels using a locally established naming convention for unique contributor sample numbers. At the admin station, medical officers used 2D scanners from their units’ Medical Communications for Combat Casualty Care sets to scan each patient’s Common Access Card (CAC) and auto-populate their DoD ID into the pooled testing label excel tracker, which matched the DoD ID to the next available specimen label. The authors felt that this approach was more accurate and efficient than typing out DoD IDs individually or finding a preassigned label for each patient on their arrival.

**FIGURE 2.**
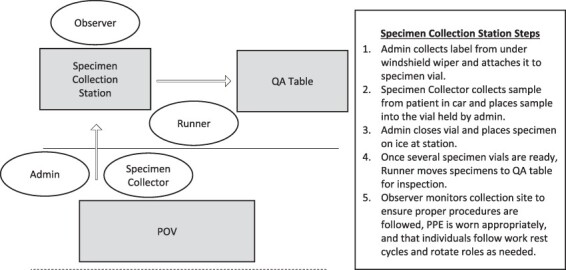
Specimen collection station roles and responsibilities. Each medic completed a standardized block of instruction from IACH’s Education Department and demonstrated competence in each of the 4 roles of specimen collector, observer, admin, and runner prior to operating independently. Each medic stayed with their specimen collection team throughout the mission but rotated through each role as the team saw fit. POV—privately owned vehicle. CAC—common access card. QA—quality assurance. PPE—personal protective equipment.

Each of the 4 specimen collection sites had a four-person team that followed standardized steps to correctly and consistently collect patient specimens (Fig. 2). QA table personnel inspected and sorted individual specimen vials into their appropriate pooled testing specimen bags. Runners then transported the pooled specimen bags to the printer area, added the pooled testing label excel tracker to the specimen bag, and turned in the pooled specimen bag to IACH lab.

### Packaging and Shipping

IACH lab worked closely with APG to understand the HQDA submission requirements for pooled specimen samples.^8^ IACH lab initially placed specimen bags upright, with each specimen individually bagged prior to placement in the pooled testing specimen bag. APG then requested that all 10 individual specimens be placed in one specimen bag together to decrease their processing time. Pooled testing specimen bags were shipped in a 16 × 14 × 14 in container with Styrofoam insert for insulation. Ice packs and disposable underpads (“chux pads”) were placed above, below, and between each specimen bag. The container was then filled with bubble wrap to minimize empty space and shifting of specimens.

APG recommended that specimens arrive with an internal temperature of 2–8 °C, although they were willing to process and certify tests with an internal temperature up to 10 °C. Under the FDA EUA for Corning VTM, specimens require refrigeration throughout, and the results may be affected by freeze-thaw cycles if the VTM freezes. APG checked and reported the temperature at the top and bottom of each shipping container upon arrival. All specimens arrived within the acceptable temperature range and were processed by APG.

IACH lab used a commercial shipping company to ensure that shipments arrived within the required 72 hours after collection. IACH lab anticipated the effects of the summer weather and weekend shipping delays with the company when determining how many ice packs and dry ice to use with each shipment. For a weekend collection that arrived at AGP 3 days after collection due to commercial shipping delays, IACH lab used 30 pounds of dried ice to ensure that the shipment arrived within standard temperature range and was able to be processed.

## RESULTS

1ID and IACH performed pooled testing collection and shipment of 2,684 specimens divided into 298 pools over 6 flight manifests. Of the 298 pooled specimens, APG found 3 to be inconclusive (SARS-CoV-2 was detected but could not be confirmed; this result should be presumed to be positive) and 1 to be invalid (no human cells were detected within the sample, and no virus was detected within the sample. Sampling procedure possibly failed to sample the individuals properly). The total number of failed pooled specimens was 4 of 298 (1.34%). The other 294 (98.7%) pooled specimens had sufficient number of human cells to be certified as COVID-19 “positive” or COVID-19 “not detected” (Table II).

**TABLE II.** Number of Sufficient, Inconclusive, and Invalid Pooled Specimen Results by Manifest Flight

Pooled specimen results by manifest flight			
Manifest flight	# Sufficient	# Inconclusive	# Invalid
ADVON	44	1	0
MB1	60	1	0
MB2	50	0	0
MB3	59	0	0
MB4	71	1	1
TRAIL	10	0	0

Abbreviation: MB—main body.

IACH and 1ID conducted follow-on individual clinical COVID-19 testing of all Soldiers with a positive, inconclusive, or invalid pooled testing result. The total number of positive results are omitted from this publication in accordance with the Secretary of Defense guidance.^9^

While the authors did not identify a published acceptable standard for the number of inconclusive and invalid results from shipped pooled testing, they feel that having an inconclusive and invalid rate of 1.34% is exceptional given the complexity of the process and condensed planning timeframe. Key lessons learned during this process are listed below.

### Standardized Training and Personnel Continuity

Overall, IACH and 1ID ensured that the same personnel were present throughout the duration of specimen collection operations. All individuals received standardized training on their specific role in the collection process, and teams maintained the same personnel. This led to extremely effective and efficient teamwork, which minimized human errors while successfully collecting up to 250 specimens per hour.

### Quality Assurance

Manning and empowering a QA table was key to identifying and correcting issues with specimens prior to submission to the lab. IACH and 1ID used a senior medical non-commissioned officer to manage this step. The QA table was closely located to all specimen collection areas and the admin area, which enabled the QA team to quickly correct any systems issues and identify the need for individual retraining.

### Administrative Oversight by Units

COVID-19 testing was a new addition to normal manifest preparation and operations. 1ID medical personnel sent the pooled testing label excel trackers from the admin station to unit leadership to track which Soldiers in each unit were tested. Although each unit had assigned times to report to the IACH specimen collection site, specimen collection throughput was frequently sporadic. This reflected the difficulty that units had with disseminating information quickly and enforcing accurate report times. The few units that set up a leadership check-in station at the collection site relayed information about compliance in real time and quickly corrected any issues with Soldiers reporting at the appropriate time.

### Collaboration and Communication

IACH and the 1ID subordinate units collectively established the pooled testing specimen collection operations. Key medical leaders were on site and actively assisting with daily operations and AARs at the end of each day’s testing. This enabled IACH and 1ID to quickly adjust tactics, techniques, and procedures in real time and relay any lessons learned in the AARs back to the entire specimen collection team and subordinate units prior to the next morning’s specimen collection.

## CONCLUSION

Pooled testing may continue to be a requirement for mass screening of COVID-19 prior to military operations. It is an effective option for units and MTFs to comply with screening requirements while conserving resources if they can establish processes to minimize human error. IACH and 1ID successfully completed pooled testing collection and shipment using the above method and would recommend a similar consolidated approach when conducting pooled testing prior to a large-scale exercise involving multiple units.

### Discussion

Planning factors may require adjustment to the above process. For example, IACH and 1ID chose a drive-through approach based on already-established processes, a large patient population that comprised multiple command and control nodes, and a manifest roster that frequently changed. If pooled testing only involved a single unit with a set roster, those patients could be preassigned a batch number and specific reporting order, eliminating the need for mass scanning of CACs on arrival.

IACH and 1ID identified a friction point in relying on a commercial shipping company to transport specimens to APG. The company did not transport specimens over the weekend. This drove the decision to collect most specimens during normal duty days, as well as required IACH lab to factor in weather’s effects on any shipping delays. An alternate approach that was not explored was to coordinate military air transportation of specimens to APG. This may also be a more feasible solution for remote areas that do not have the ability to coordinate overnight shipping to APG through commercial shipping companies.

IACH and 1ID were constrained by the inability to pool the individual specimens and conduct the laboratory testing of pooled specimens at IACH. This made operations more complex and required specimen shipment to APG, but IACH was able to preserve its PCR testing capabilities to maintain local COVID-19 mitigation operations and conduct individual retests of patients from positive, inconclusive, or invalid pools. If the unit and MTF are able to hold all specimens while conducting pooled testing on site and conduct the follow-on individual retesting of positive, inconclusive, or invalid pools, it would simplify the process from a shipping standard. However, many additional planning factors and potential obstacles would need to be considered to run a large number of pooled tests at local MTFs.

Similarly, Army Public Health Center—Laboratory Sciences at APG did not perform individual retesting of positive pools and instead required that it be done in a clinical setting. If APG was able to preserve all specimens while conducting pooled testing and conduct the follow-on individual retesting of positive, inconclusive, or invalid pools using the original specimens, it could significantly decrease the number of patients who need to have samples recollected. However, there are numerous planning factors and potential obstacles that would need to be considered to maintain specimens and retest individual specimens using clinical setting standards.

Medical planners should consider disease incidence, local testing capability and competing priorities, and deployment timeline when influencing the decision to request pooled testing instead of individual testing to screen individuals before military operations. The spread of the delta variant may contribute to a higher disease incidence, which would result in a larger number of positive pools and an increased workload to conduct follow-on individual retesting. Local MTFs must utilize medical supplies and testing capabilities for that retesting. Units must ensure that Soldiers get retested as needed and wait on that retesting to determine if those individuals are clear to deploy with the unit. In some scenarios, individual screening of all patients at the local MTF can be conducted in a faster and more streamlined process.

1ID and IACH were not required to continue pooled specimen testing after 1ID’s CTC rotation, and they reverted back to in-house clinical testing of individuals using the IACH drive-through testing site for subsequent operations. If pooled testing is required or requested in the future, the authors recommend using the above processes. Key factors for pooled testing to be successful in any situation are standardized training and personnel continuity, quality assurance, administrative oversight by the unit, and collaboration and communication between all involved entities.
